# Risk Factors for Hospital Admissions Among Emergency Department Patients: From Triage to Admission

**DOI:** 10.5811/westjem.21263

**Published:** 2025-02-25

**Authors:** Jemima Koh, Oh Hong Choon, Seah Zeyen, Steven Lim

**Affiliations:** * Health Services Research, Changi General Hospital, Singapore; † Changi General Hospital, Department of Emergency Medicine, Singapore; ‡ Duke-NUS Medical School, Health Services and Systems Research, Singapore; § Centre for Population Health Research and Implementation, Singapore Health Services Pte Ltd, Singapore

## Abstract

**Introduction:**

Healthcare systems typically provide multiple channels to access acute inpatient care, with the emergency department (ED) as the main route of access. The ED faces multifaceted demand and supply challenges, which implicate resource allocation and patient flow. In this study we aimed to identify factors associated with hospital admissions among ED patients in a Singapore tertiary-care hospital.

**Methods:**

Using a retrospective cohort study of all eligible visits to a Singapore ED between January 1–December 31, 2019, we conducted a multivariable, mixed-effect logistic regression model to study the factors associated with hospital admissions. The model accounted for patients’ demographics; triage category; arrival mode; referral source; time of ED visit; discharge diagnosis; and ED occupancy levels.

**Results:**

In 2019, there were 141,719 visits to the ED, with 42,238 (29.8%) of these visits resulting in hospital admissions. Factors associated with increased odds of hospital admissions included increasing age, being male, ethnicity (Malay vs Chinese), higher patient acuity, non-self-referred patients (vs self-referred), patient being conveyed by ambulances (vs walk-in), and category of disease. Our model demonstrated that the highest odds of inpatient admissions were attributed to the patient’s acuity (highest vs lowest acuity: odds ratio [OR] 326, 95% confidence interval [CI] 292–363), followed by patients’ age (70 and above vs 30 and below: OR 13.8, 95% CI 12.8–14.8). The ORs for all other factors with significantly increased odds of admissions were modest, ranging from 1.12–4.18. Although the ED occupancy levels at the hour of the patient’s disposition decision, the hour of the ED visit, and the month of the ED visit were significantly associated with hospital admissions, changes in the probabilities of hospital admissions across the possible range of values of these factors were marginal.

**Conclusion:**

Our study revealed several factors significantly associated with hospital admissions, with patient acuity and age as the most important factors. Moreover, emergency physicians’ decisions to admit patients were clinically consistent and only marginally influenced by the degree of ED crowding. These findings offer invaluable insights into follow-up studies that will be crucial in shaping new policies or designing new interventions to enhance current preventive health or healthcare delivery systems to curtail the growth in inpatient-bed demand among ED patients over time.

## INTRODUCTION

Well-developed healthcare systems typically provide multiple channels to access acute inpatient care, with the emergency department (ED) as the main route of access 1 The ED faces multifaceted demand and supply challenges, which implicate resource allocation, patient safety, and patient flow.^2^ Studies found that factors attributing to the increased demand for ED services include the ageing population and behavioural changes to healthcare services decisions.^3^ Amidst global ED crowding, understanding the effect of ED and inpatient-bed occupancy rates on the rate of hospital admissions is vital, given their potential negative impact on patient outcomes and department functionality.^4^–^6^

Singapore is a rapidly ageing country with an increasing number of older adults in the last decade, and this trend is expected to persist in the next decade.^7^ The ageing demographics contributed to a visible proportion of ED visits and increased the demand for many healthcare services.^8^ Local public hospital EDs see a daily attendance ranging from 250–450 patients^9^ attributed to factors such as free ambulance services,^10^ a low triage rejection rate, and the public perception associating non-acute concerns with necessary specialist care.

Changi General Hospital (CGH) is a public, tertiary-care hospital located in the eastern part of Singapore, serving the regions with the highest proportion of older adults.^11^,^12^ Its ED is also one of the busiest in the country, with at least 35% of 2019 having a high load of daily attendances of >400 patients. Over the last few years, while attendance at CGH ED observed a relatively consistent trend, the hospital admission rate among ED patients had increased from 22.6% in financial year (FY) 2012 to 30.2% in FY 2019. Research has shown that admission from the ED is dependent on several factors such as patient profile, acuity levels, diagnosis, and arrival patterns.^1^,^2^ While much is known about the factors associated with hospital admissions in Western countries for specific groups of patients,^13^–^17^ little is known about what affects admissions in a densely populated Asian country like Singapore, which faces the challenge of a swelling geriatric population. In this study, therefore, we aimed to identify the factors associated with hospital admissions in CGH. Considering the shift in the rate of hospital admission, it is imperative to understand and identify the factors associated with hospital admissions. These findings can offer insights that can potentially enhance the current healthcare systems in hospitals so that the demand for inpatient beds can be better managed in the future.

In this study we accounted for several factors that were less commonly explored in the existing literature, where reviews of the association with hospital admissions were mixed and inconclusive. In addition to patient demographics, ED diagnoses and arrival modes, we also studied the effect of ED occupancy levels and the timing of patient arrival at the ED.

## METHODS

### Study Design, Setting and Participants

This was a retrospective study of visits that took place at the ED of CGH between January 1–December 31, 2019. We obtained data from the administrative databases of the hospital ED.

### Data Sources and Variables

The outcome of the study, hospital admission, defined as the inpatient admission that follows an ED attendance, was obtained from the patient’s discharge type. In our dataset, patients’ discharge type was categorised into many categories (eg, treated and discharged, admitted to the ward, referred to general practitioners). Hospital admission was defined as an outcome if the ED patient was subsequently admitted to an inpatient ward (excluding short-stay units [SSU] in the hospital, where the SSU is a ward within the ED for observation of ED patients with specific clinical conditions and do not require inpatient admissions for up to 23 hours).

**Population Health Research Capsule**

What do we already know about this issue?

*Patient profiles and arrival traits affect emergency department (ED) admissions in Western countries, but the impact of ED patient volume is mixed.*

What was the research question?

*We sought to identify factors associated with hospital admissions among ED patients in Singapore.*

What was the major finding of the study?

*Hospital admissions via the ED (29.8% of all ED patients) were associated with increasing age and higher clinical acuity, but were marginally affected by ED patient volume.*

How does this improve population health?

*These findings could help shape policies and interventions to improve healthcare delivery systems and reduce inpatient bed demand among ED patients over time.*

We identified independent variables from published studies and discussions with domain experts within the hospital. We included patient characteristics (age, sex, ethnicity), discharge diagnosis based on the International Classification of Diseases Revision 10 (ICD-10) codes, triage level based on acuity, arrival characteristics (mode of arrival, source of referral to the ED, hour of arrival, day of the week, and month of the year of ED visit), as well as ED occupancy level.

For this study we grouped patients by age as follows: ≤30 year of age; ≥71; and age groups of 10-year intervals between ages 31–70. (Results for a separate model where age was modelled non-lineary is presented in the appendix. The patients’ sex was categorised as male or female, and ethnicity was categorised as Chinese, Malay, Indian, or other. The patients’ mode of arrival to the ED was characterised as walk-in or arrival by any type of ambulance. Patients could be referred to the ED by themselves (self-referral), intermediate and long-term care (ILTC) service professionals (eg, community hospitals, nursing homes), primary care physicians, government agencies (eg, police station or prison), or other. The patients’ three-character ICD-10 codes were grouped into 10 broad ICD categories following the ICD-10 2019. 18 We extracted the 22 overarching categories from the ICD-10 and identified our hospital’s nine most commonly diagnosed ICD-10 codes. The ICD-10 codes (apart from the nine identified) were grouped under “other” due to their lower frequencies. Acuity levels were categorised into P1, P2, and P3 where P1 represents the highest acuity for trauma patients with life-threatening conditions and P3 for patients with the lowest acuity, without urgent treatments or procedures required.^19^ As there was no P4 acuity presented in the year of study, we excluded P4. We obtained ED arrival times by the hour, day of the week, and month of the year from patients’ admission timestamps recorded when patients registered upon arrival.

The ED occupancy (or census) level was used as a representation of the degree of crowding in the ED. This variable represents the number of patients being cared in by the ED (inclusive of patients in the waiting room), also known as the number of patients in the ED at any time point in the study period, and it was derived using the respective times of arrival to discharge times of all patients. To understand the impact of the degree of crowding on admission decisions, the number of patients in the ED was matched to the hour of the emergency physician’s final disposition decision regarding the patient (ie, to admit or discharge the patient) after reviewing relevant results. This variable serves as a proxy to gauge the ED occupancy level to perceive the crowded conditions on the floor.

### Statistical Methods

We summarised characteristics of patients and their visits in our study population with mean and standard deviation or frequency with percentage for continuous and categorical variables, respectively. We conducted a comparison of these characteristics among these patients, with and without the outcome, using a two-sample *t*-test and chi-square test for continuous and categorical variables, respectively.

As patients could visit the ED more than once in 2019, we conducted a multivariable, mixed-effects logistic regression with random intercepts by patients to study the factors associated with urgent admission from the ED.^20^ We fitted an unstructured covariance structure to account for the correlation between repeat patients in the dataset.^21^ Candidate factors were identified from published studies and discussions with domain experts. We chose the final model presented in this study based on likelihood ratio tests.^22^ Factors in the final model were either non-linear variables (ED occupancy levels, hour of admission to the ED, and the month of year) or categorical variables (all others).

We studied non-linear variables using restricted cubic splines (RCS) with a suitable number of knots placed at relevant quantiles, as recommended by Harrell.^22^ For the month of arrival, we fitted a 3-knot RCS at the 10^th^, 50^th^, and 90^th^ quantile of the data. For the ED occupancy levels and the hour of the day, we fitted a 4-knot RCS at the 5^th^, 35^th^, 65^th^, and 95^th^ quantile of the data.^22^ We assessed associations between the non-linear variables and the outcome using a likelihood ratio test of a model with and without the RCS. 22

We present the odds ratio (OR) with a 95% confidence interval (CI) for the association between categorical variables with the outcome. Reference categories for variables were selected based on their descriptive distributions (choosing categories with the largest proportion for nominal data or those with the lowest likelihood of outcome for ordinal data) or based on clinical judgement (wherein clinicians identified the most appropriate reference category for comparisons). While RCS models are flexible and better suited for modelling non-linear data, the regression coefficients are hard to interpret. Therefore, for non-linear variables, we illustrated the relationship with the outcome by predicting the probability of hospital admission for each non-linear variable on an exemplar patient. The exemplar patient was defined based on values of each factor that had the highest proportion of hospital admission and was characterised by its 95% prediction intervals.

Statistical significance for this study was set at a 5% level. All data processing and statistical analysis were conducted in R version 4.1.1. (R Foundation for Statistical Computing, Vienna, Austria).^23^ We used the packages lme4 24 and rms 25 to build the mixed effect model and the model the restricted cubic splines respectively. The respective functions were glmer and rcs. To strengthen the report of our study, we adhered to the items in the STROBE statement checklist. 26

### Ethics Approval

This study was reviewed by the SingHealth Centralized Institutional Review Board, which determined that this study did not require further ethical deliberation because it was a service evaluation project aiming to study factors associated with hospital admissions from ED.

## RESULTS

### Participants

In 2019 there was a total of 144,136 visits from 99,700 patients. We excluded from the study 422 visits without discharge outcomes (0.3%), two with undefined sex (0.0%), and 1,975 (1.4%) with incomplete timestamps, resulting in 141,719 visits from 98,558 participants to analyze.

### Outcome Data and Overall Descriptive Data

Amongst the 141,719 visits to the ED, 30% resulted in hospital admissions in 2019 (Table 1). Half of the visits were made by patients >50 years old, and a significantly higher proportion of hospital admissions was seen in the older age groups; 59% of the patients were male, with a higher proportion of admission seen amongst males compared to females; and 52% of the patients were Chinese, followed by Malays (21%), other (14%), and Indian (12%). There was a higher proportion of Chinese who were admitted compared to those who were not. The largest proportion of ICD-10 diagnoses presented at the ED were symptoms, signs, and abnormal clinical and laboratory findings, not elsewhere classified (21%). Furthermore, 49% of patients were classified as P2, and 38% as P3, while 81% of patients walked into the ED. There was a higher proportion of hospital admission amongst those arriving by ambulance. The proportion of visits on various days of the week was slightly higher on Mondays (17%) and Tuesdays (15%), and there was a similar proportion of visits across different months of the year (8–9%).

### Main Results

The multivariable, logistic mixed-model results showed that older patients had increasing odds of hospital admissions (Table 2). Patients 31–40 year of age had 1.28 times (95% CI 1.19–1.38) while those ≥71 years of age had 13.8 times (95% CI 12.8–14.8) higher odds of hospital admissions as compared to patients <30. As compared to males, females had significantly lower odds of hospital admissions by 0.949 times (95% CI 0.914–0.986). We also found that Malays (OR 1.12, 95% CI 1.07–1.18) and other races (OR 1.19, 95% CI 1.12–1.26) had significantly higher odds of hospital admission compared to Chinese patients.

We observed that the days of the week did not affect the odds of hospital admission (compared to Wednesdays). Patients who arrived by ambulance had significantly higher odds of hospital admission by 1.64 times (95% CI 1.57–1.72) compared to those who walked into the ED. Patients who were referred to the ED by any form of referral source (government agency (OR 1.15, 95% CI 1.04–1.28), ILTC professionals (OR 1.79, 95% CI 1.50–2.12), primary care physicians (OR 1.85, 95% CI 1.76–1.94]), and others (OR 4.18, 95% CI 3.55–4.93]) also had significantly higher odds of hospital admission compared to self-referral. As compared to patients with the lowest acuity (P3), those with higher acuities had significantly higher odds of hospital admission. As compared to patients with diseases of the respiratory system, those with diseases of the circulatory system (OR 2.01, 95% CI 1.83–2.20), diseases of the digestive system (OR 2.17, 95 CI 1.98–2.37), and diseases of the skin and subcutaneous tissue (OR 2.14, 95% CI 1.94–2.36) had significantly higher odds of hospital admission, while those with diseases of the musculoskeletal system and connective tissue (OR 0.428, 95% CI 0.388–0.472), “injury, poisoning and certain other consequences of external causes” (OR 0.238; 95% CI 0.219–0.258), “symptoms, signs and abnormal clinical and laboratory findings, not elsewhere classified” (OR 0.866, 95% CI 0.806–0.93), and other ICD-10 categories (OR 0.729, 95% CI 0.674–0.789]) had significantly lower odds of hospital admission.

We studied the relationship between non-linear variables and hospital admission by illustrating the probability of hospital admission for an exemplary patient; we predicted the probabilities for the range of the variable in the dataset. Specifically, we predicted probabilities for a male, Chinese patient >70 years of age, who walked into the ED by himself. The patient presented with an acuity of P2 and was diagnosed with ICD-10 code “symptoms, signs and abnormal clinical and laboratory findings, not elsewhere classified.” The patient came on a Monday in July at 10 am when the number of patients in the ED was 100.

Figure 1 illustrates the relationship between the adjusted probability of hospital admission and the non-linear variables. At different ED occupancy levels (Figure 1A), the lowest probability of hospital admission (63%) was at levels of fewer than 48 ED patients in the ED. Between 48–174 patients in the ED, the probability of admission was around 65%, before increasing to 71% when the number of patients in the ED was above 174 (*P* = 0.04). At different hours of arrival to the ED (Figure 1B), the probability of hospital admission varied marginally over a range of 62–69%, with the highest probability of admission at 20 hours, and the lowest at 0 hours (*P* <0.05). At different months of the year (Figure 1C), there was a marginal increase from January to December where the probability of hospital admission ranged from 66–71% (*P* <0.05).

## DISCUSSION

### Key Results

Our study identified several factors associated with higher odds of hospital admission. We found that older patients; males; Malay patients and those of other ethnicity (compared to Chinese); patients with diseases of the circulatory system, diseases of the digestive system, diseases of the skin and subcutaneous tissues (as compared to diseases of the respiratory system); higher acuity categories; non-self-referral (compared to self-referral); and arrival by ambulance (compared to walk-ins) had significantly higher odds of urgent admission.

Although the ED occupancy levels at the hour of the patient’s disposition decision, hour of ED visit, and month of ED visit were significantly associated with hospital admission, the changes in the probability of hospital admission were marginal. A slightly higher probability of hospital admission was noted at a higher number of patients in the ED, post-evening hours, and at the end of the year.

### Interpretation

We found that the factors affecting patient admissions were consistent with the findings of a larger study conducted across 19 EDs.^27^ Older males with a higher number of comorbid conditions and patients presenting during the evening or night shifts were significantly more likely to be admitted. These factors were similar to the results of our study. Another study in the United States revealed that ED visits on a Monday were associated with a longer duration of ED length of stay (LOS).^28^ The authors hypothesised that the nation’s EDs might be experiencing a resource shortage on typical Mondays, whereby demand outweighs supply, contributing to longer ED stays. Using ED LOS as a proxy for the volume of ED patients, it was hypothesized that longer ED LOS and a higher patient load in the ED would result in higher odds of admission, particularly on a Monday.

Contrary to these findings, we found that the odds of admission were not influenced by the day of the week. This could be due to several admission-avoidance strategies, including the setup of a SSU ward in ED for observation (up to 23 hours) of ED patients who have specific clinical conditions and do not require inpatient admission; the practice of observational medicine^29^ among ED staff; accessibility of primary care physicians even on weekends; and availability of fast-track specialist clinics for eligible ED patients. Additionally, the CGH ED uses a demand-driven optimal staffing approach to manage patient influx.^30^ Specifically, the ED allocates more manpower on Mondays and Tuesdays compared to the rest of the week since the likelihood of recalling or activating off-duty personnel when unforeseen circumstances occur on Mondays and Tuesdays is higher. This maintains adequate staffing support in the ED to handle the patient load without compromising admission decisions.

We also found ED occupancy levels to be only marginally associated with admissions. We observed marginally higher odds of admission when a greater number of patients were present in the ED. One plausible rationale for this relationship is the indirect reflection of physician workload.^4^ In a crowded ED, emergency clinicians face a heavier workload. Workup for safe discharge can be time-consuming and resource-intensive. Given these constraints, physicians might opt for a more conservative approach by admitting an ED patient whenever there is doubt about the patient’s condition. A retrospective analysis by Wyatt et al provided differing perspectives as to how the non-clinical factors in their ED environment affected the rate of admission. 5 As the ED occupancy level increases, the staff-to-patient ratio decreases and the time taken to care for each patient in turn becomes longer. As the LOS of these patients extends, fortunately, they are still within the ED when the results of their diagnostic tests become available. This allows for a more comprehensive assessment of their condition, potentially leading to the conclusion that they are safe for discharge. Additionally, in some cases, the nature of their symptoms may spontaneously improve, reducing the need for hospitalization.

In a qualitative study conducted by Pope et al they interviewed ED and inpatient staff to identify other key factors affecting hospital admissions among ED patients.^31^ They found a significant influence on departmental culture and the personality of the physician in charge. These aspects set the risk appetite and create benchmarks for admission vs discharge decisions for the patients who present to the ED during a particular shift. Additionally, some physicians are more inclined to admit patients as a response to patients’ preferences and expectations when there might not be clinical indications for doing so. Although such nuances are difficult to capture in quantitative studies, these findings underscore the complexity of the decision-making process in the ED, where physicians must balance patient expectations with adherence to clinical best practices when making hospital admission decisions.

Many studies have looked at the factors associated with hospital admissions focused on a specific group of patients with certain characteristics or medical conditions, with the proportions of admissions to hospitals from the ED general patient population not widely reported publicly in many countries.^13^,^14^,^16^,^32^,^33^ However, amongst a few countries with such statistics, we saw that in 2021 it was reported that 13.1% of emergency visits in the United States resulted in hospital admission, a lower percentage compared to our study. 34 Despite the difference in proportion, the US researchers found that visits from older patients and those from nursing homes, or who arrived by ambulance and those with higher triage categories had higher percentages of admissions, similar to our findings. Another large study done in 1,375 EDs in the US reported admission rates varying from 9.8–25.8% and found that higher ED admission rates were linked with more Medicare or uninsured patients, more beds, lower ED volume, for-profit ownership, trauma center status, and higher occupancy rates.^35^

While the reasons driving these differences are complex, some of the possible reasons for discrepancies in hospital admission rates could arise from differences in healthcare systems. For example, the prerequisite that an ED visit in Denmark has to be initiated through a general practitioner’s referral may potentially reduce the number of ED visits and subsequent hospital admissions.^36^ Other contributing factors include variations in payment structures, such as subsidized fees or charges associated with emergency care services. Other possible reasons could also include admission criteria, patient population and demographics, and access to primary care, as well as public awareness and education regarding when to seek emergency advice.

Knowing the diseases, ethnicities, diseases and age groups with an increased chance of hospital admission can inform and tailor preventive health-related interventions to target specific populations in the region at the primary care level. Local initiatives that tap community-based clinicians to manage some acute conditions, empowering general practitioners (GP) and encouraging patients to follow up on chronic conditions with their primary care physicians have been useful in improving patient flow. Supplementing these initiatives can help curtail the rising inpatient bed demand in the future if conditions are managed at the community level. An example of such initiative is the GPFirst programme, introduced in CGH in 2014, where patients were given S$50 subsidies for their ED attendance if they were referred to the CGH ED by participating GPs.^37^ This program encourages patients to visit their GPs first instead of going to the ED if their conditions are non-urgent or mild. Our analyses provide insights to direct the expansion of these programs by identifying the factors associated with increased hospital admission.

### Strengths

There are several strengths to our study. Firstly, the sample size was large. This allowed the study team to focus on the effect size of the factors associated with the outcome as statistical significance was readily achieved. We obtained data from administrative databases that were not subjected to recall or observation bias, which we believe enhanced the quality of the study. We were, thus, able to control for several factors due to the quality of our administrative dataset. In this study, we reviewed less impactful factors such as ED occupancy levels at the hour of the patient’s disposition decision, triage level, diagnosis, arrival mode, and referral sources.

## LIMITATIONS

As a single-centre, retrospective study, our findings may not apply to other local hospitals serving a different patient demographic. We used data from 2019 before COVID-19 impacted and altered the systems and dynamics of healthcare services in hospitals. Future research from 2022 should be considered when the situation in EDs has stabilised from the impact of the pandemic. We used ICD-10 diagnoses according to the ED visit, which may differ at the point of inpatient discharge. Due to data limitations, we did not account for socioeconomic factors and behaviours associated with hospital admission^17^; nor were we able to explore how a physician’s behaviour during the patient’s deposition decision affects outcomes. Lastly, although there could be a possible interplay between hospital admission and inpatient units, which could be explored by looking at the inpatient bed occupancy rate (as discussed by Wyatt et al^5^), it was not feasible in this study to analyze this dynamic due to data constraints.

## CONCLUSION

To the best of our knowledge, this is the first study in Singapore to identify factors associated with hospital admissions. We saw that the strongest association with hospital admission was patient acuity and age. Overall, emergency physicians’ decisions to admit patients were clinically consistent and only marginally influenced by the degree of ED crowding. These findings offer invaluable insights into possible follow-up studies that will be crucial in shaping new policies or designing new interventions that aim to enhance preventive health or healthcare delivery systems, so that the growth in demand for inpatient beds for ED patients can be curtailed over time.

## Supplementary Information



## Figures and Tables

**Figure:**
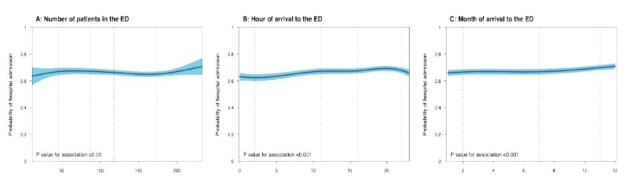
Probability of hospital admission for non-linear variables. Predicted probability of hospital admission for an exemplary patient; a male, Chinese patient > 70 years of age, who walked into the ED by himself. The patient presented with an acuity of P2 and was diagnosed with ICD-10 code “Symptoms, signs and abnormal clinical and laboratory findings, not elsewhere classified.” The patient came on a Monday of the month of July at 10 AM when the number of patients in the ED was 100. The P-value for association was tested with a likelihood ratio test of a model with and without the spline function. Vertical dotted lines of each plot represent the knots placed at relevant quantiles, with 4 knots fitted at the 5th-, 35th, 65th, and 95th-quantile of the data for the ED occupancy levels and a 3 knots fitted at the 10th-, 50th- and 90th-quantile of the data for the hour of arrival to the ED and the month of arrival to the ED. The blue-shaded regions represent the 95% predicted intervals, and the P-value for the association tested with a likelihood ratio test.

**Table 1:** Overall characteristics of patients and their visits included in the study.

	Overall (N=141,719)	Hospital admission		P-value^1^

		Yes (n=42,238, 29.8%)	No (n=99,481, 70.2%)	
Age (continuous), mean (SD)	49.3 (22.0)	64.1 (19.3)	43.0 (20.0)	<0.001
Age (categorical), N (%)				
≤30	38,219 (27%)	3,139 (7%)	35,080 (35%)	<0.001
31 to 40	19,615 (14%)	2,817 (7%)	16,798 (17%)	
41 to 50	15,823 (11%)	3,647 (9%)	12,176 (12%)	
51 to 60	19,759 (14%)	6,531 (15%)	13,228 (13%)	
61 to 70	19,745 (14%)	8,332 (20%)	11,413 (11%)	
≥71	28,558 (20%)	17,772 (42%)	10,786 (11%)	
Sex, N (%)				
Female	57,985 (41%)	18,511 (44%)	39,474 (40%)	<0.001
Male	83,734 (59%)	23,727 (56%)	60,007 (60%)	
Ethnicity, N (%)				
Chinese	74,344 (52%)	24,555 (58%)	49,789 (50%)	<0.001
Malay	30,469 (21%)	88,94 (21%)	21,575 (22%)	
Indian	16,634 (12%)	3,901 (9%)	12,733 (13%)	
Other	20,272 (14%)	4,888 (12%)	15,384 (15%)	
ICD-10 categories, N (%)				
Certain infectious and parasitic diseases	11,969 (8%)	2,980 (7%)	8,989 (9%)	<0.001
Diseases of the circulatory system	7,911 (6%)	5,481 (13%)	2,430 (2%)	
Diseases of the digestive system	8,305 (6%)	3,683 (9%)	4,622 (5%)	
Diseases of the genitourinary system	6,206 (4%)	2,144 (5%)	4,062 (4%)	
Diseases of the musculoskeletal system and connective tissue	13,468 (10%)	1,417 (3%)	12,051 (12%)	
Diseases of the respiratory system	15,179 (11%)	4,794 (11%)	10,385 (10%)	
Diseases of the skin and subcutaneous tissue	6,172 (4%)	2,550 (6%)	3,622 (4%)	
Injury, poisoning and certain other consequences of external causes	24,059 (17%)	2,838 (7%)	21,221 (21%)	
Symptoms, signs and abnormal clinical and laboratory findings, not elsewhere classified	29,966 (21%)	10,635 (25%)	19,331 (19%)	
Others	18,484 (13%)	5,716 (14%)	12,768 (13%)	
Patient acuity, N (%)				
P1 (highest acuity)	18,823 (13%)	15,248 (36%)	3,575 (4%)	<0.001
P2	68,962 (49%)	26,133 (62%)	42,829 (43%)	
P3 (lowest acuity)	53,934 (38%)	857 (2%)	53,077 (53%)	
Mode of arrival, N (%)				
Walk-in	114,609 (81%)	24,970 (59%)	89,639 (90%)	<0.001
By ambulance	27,110 (19%)	17,268 (41%)	9,842 (10%)	
Source of referral, N (%)				
Self-referral	104,855 (74%)	30,090 (71%)	74,765 (75%)	<0.001
Primary care	28,806 (20%)	7,797 (18%)	21,009 (21%)	
ILTC^2^	1,993 (1%)	1,632 (4%)	361 (0%)	
Government agency^3^	4,673 (3%)	1,828 (4%)	2,845 (3%)	
Others	1,392 (1%)	891 (2%)	501 (1%)	
Arrival hour, N (%)				
07:00–12:00	46,077 (33%)	13,838 (33%)	32,239 (32%)	<0.001
13:00–21:00	67,617 (48%)	20,837 (49%)	46,780 (47%)	
22:00–06:00	28,025 (20%)	7,563 (18%)	20,462 (21%)	
Number of patients in the ED, mean (SD)	105 (39.1)	107 (39.1)	104 (39.1)	

1 Comparisons between two groups were done using a chi-square test for categorical variables.

2 Intermediate and long-term care (ILTC) providers (eg, community hospitals and nursing homes).

3 Government agency (e.g., police station or prison).

*ILTC*, intermediate and long-term care; *ICD-10*, International Classification of Diseases, 10th Rev.

*ED*, emergency department; *SD*, standard deviation.

**Table 2:** Odds ratio with 95% confidence intervals from mixed-effect, multivariable logistic regression^1^ for factors associated with hospital admission.

Variables	Categories	OR [95% CI]	P-value
Age group (reference ≤30)	31 to 40	1.28 [1.19, 1.38]^*^	<0.01
	41 to 50	1.94 [1.81, 2.08]^*^	<0.01
	51 to 60	2.68 [2.51, 2.87]^*^	<0.01
	61 to 70	3.71 [3.47, 3.96]^*^	<0.01
	≥71	13.8 [12.8, 14.8]^*^	<0.01
Sex (reference male)	Female	0.949 [0.914, 0.986]^*^	<0.01
Ethnicity (reference Chinese)	Malay	1.12 [1.07, 1.18]^*^	<0.01
	Indian	0.958 [0.9, 1.02]	0.184
	Others	1.19 [1.12, 1.26]^*^	<0.01
Day of ED visit (reference Wednesday)	Monday	1.04 [0.972, 1.11]	0.261
	Tuesday	1.02 [0.954, 1.09]	0.569
	Thursday	0.956 [0.895, 1.02]	0.176
	Friday	0.978 [0.915, 1.05]	0.507
	Saturday	0.997 [0.927, 1.07]	0.945
	Sunday	1.03 [0.96, 1.11]	0.401
Arrival mode (reference walk-in)	By ambulance	1.64 [1.57, 1.72]^*^	<0.01
Source of referral (reference self-referral)	Government agency	1.15 [1.04, 1.28]^*^	<0.01
	ILTC	1.79 [1.5, 2.12]^*^	<0.01
	Primary care	1.85 [1.76, 1.94]^*^	<0.01
	Others	4.18 [3.55, 4.93]^*^	<0.01
Acuity [reference P3 (lowest acuity)]	P1 (Highest acuity)	326 [292, 363]^*^	<0.01
	P2	54.8 [50.2, 59.7]^*^	<0.01
CD-10 categories (reference diseases of the respiratory system)	Certain infectious and parasitic diseases	1.06 [0.972, 1.17]	0.176
	Diseases of the circulatory system	2.01 [1.83, 2.2]^*^	<0.01
	Diseases of the digestive system	2.17 [1.98, 2.37]^*^	<0.01
	Diseases of the genitourinary system	0.932 [0.843, 1.03]	0.164
	Diseases of the musculoskeletal system and connective tissue	0.428 [0.388, 0.472]^*^	<0.01
	Diseases of the skin and subcutaneous tissue	2.14 [1.94, 2.36]^*^	<0.01
	Injury, poisoning and certain other consequences of external causes	0.238 [0.219, 0.258]^*^	<0.01
	Others	0.866 [0.806, 0.93]^*^	<0.01
	Symptoms, signs and abnormal clinical and laboratory findings, not elsewhere classified	0.729 [0.674, 0.789]^*^	<0.01

1 Multivariable mixed-effect logistic regression with random intercepts by patients and an unstructured covariance structure to account for the correlation beween repeated patients in the dataset.

*CI*, confidence interval; *ED*, emergency department; *ILTC*, intermediate and long-term care; *ICD-10*, International Classification of Diseases, 10th Rev.
