# Supplementary material for: Risk Factors for Hospital Admissions Among Emergency Department Patients: From Triage to Admission
Source: West J Emerg Med. 2025 Feb 25;26(3):513–22. doi: 10.5811/westjem.21263 (PMC12208046; doi:10.5811/westjem.21263)
Supplement: Supplementary file 1 [file wjem-26-513-s001.docx]

**SUPPLEMENTARY**

Below details the results when age was studied as a continuous variable instead of categorical variable (as presented in the main paper)

**Appendix Table 2.** Odds ratio with 95% CI from mixed-effect multivariable logistic regression^1^ for factors associated with hospital admission with age studied continuously.

| Variables | Categories | OR [95% CI] | *P*-value |
| --- | --- | --- | --- |
| Sex Reference male | Female | 0.916 [0.883, 0.951]* | <0.01 |
| Ethnicity Reference Chinese | Malay | 1.15 [1.1, 1.21]* | <0.01 |
|  | Indian | 0.987 [0.928, 1.05] | 0.679 |
|  | Others | 1.19 [1.12, 1.26]* | <0.01 |
| Arrival mode Reference walk-in | Some form of ambulance | 1.58 [1.5, 1.65]* | <0.01 |
| Source of referral Reference self-referral | Government agency | 1.15 [1.04, 1.28]* | <0.01 |
|  | ILTC | 1.6 [1.34, 1.89]* | <0.01 |
|  | Primary care | 1.87 [1.78, 1.96]* | <0.01 |
|  | Others | 4.18 [3.55, 4.92]* | <0.01 |
| Acuity Reference P3 | P1 | 341 [306, 380]* | <0.01 |
|  | P2 | 58.5 [53.6, 63.9]* | <0.01 |
| ICD 10 categories Reference Diseases of the respiratory system | Certain infectious and parasitic diseases | 1.09 [0.992, 1.19] | 0.0756 |
|  | Diseases of the circulatory system | 2.05 [1.87, 2.24]* | <0.01 |
|  | Diseases of the digestive system | 2.17 [1.99, 2.37]* | <0.01 |
|  | Diseases of the genitourinary system | 0.934 [0.846, 1.03] | 0.177 |
|  | Diseases of the musculoskeletal system  and connective tissue | 0.443 [0.401, 0.488]* | <0.01 |
|  | Diseases of the skin and subcutaneous tissue | 2.13 [1.93, 2.35]* | <0.01 |
|  | Injury, poisoning and certain other  consequences of external causes | 0.248 [0.228, 0.269]* | <0.01 |
|  | Others | 0.744 [0.689, 0.805]* | <0.01 |
|  | Symptoms, signs and abnormal clinical and laboratory findings, not elsewhere classified | 0.877 [0.817, 0.942]* | <0.01 |

^1^Multivariable mixed-effect logistic regression adjusted for day of the week with random intercepts by patients and an unstructured covariance structure to account for the correlation between repeated patients in the dataset.

CI, confidence interval; ED, emergency department.


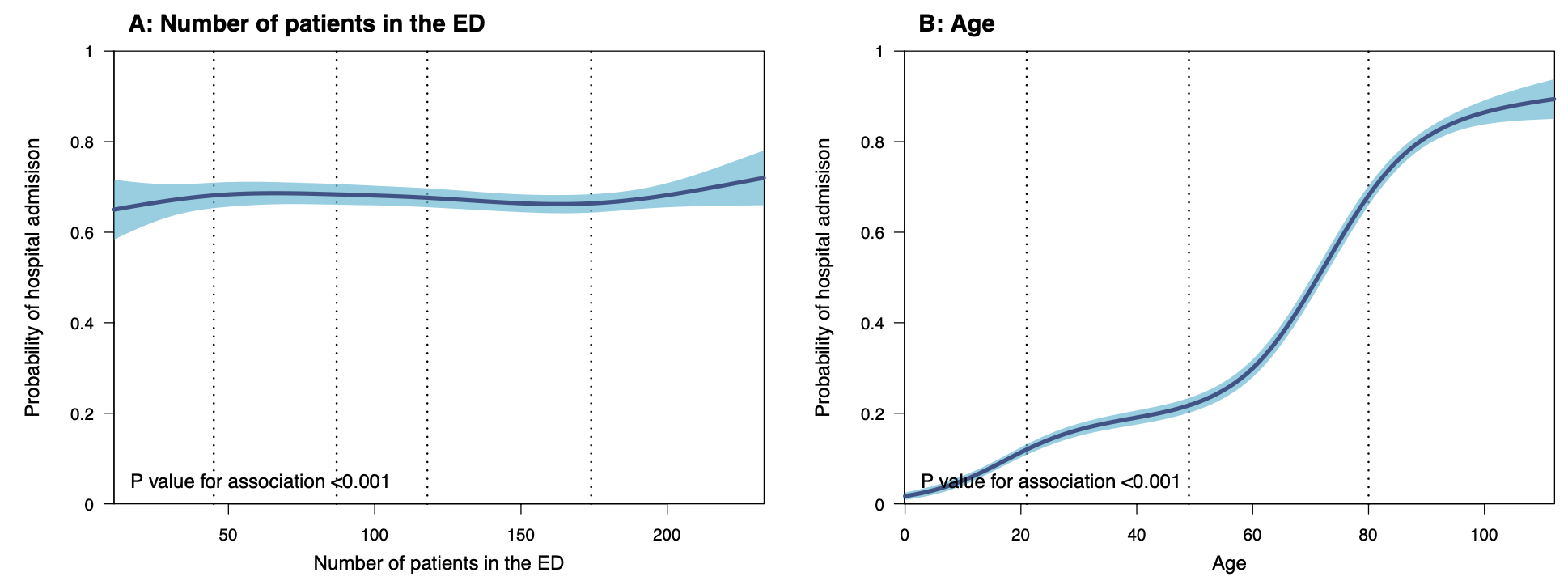


**Appendix Figure 1.** Probability of hospital admission for non-linear variables (A: Number of patients in the ED and B: Age) from a multivariable mixed-effect logistic regression with random intercepts by patients and an unstructured covariance structure to account for correlation between repeated patients in the dataset.

Predicted probability of hospital admission for an exemplary patient; a male, Chinese patient of age 80, who walked into the ED by himself. The patient presented with an acuity of P2 and was diagnosed with ICD10 code Symptoms, signs and abnormal clinical and laboratory findings, not elsewhere classified. The patient came on a Monday of the month of July at 10 am when the number of patients in the ED was 100. The P-value for association was tested with a likelihood ratio test of a model with and without the spline function. Vertical dotted lines of each plot represent the knots placed at relevant quantiles as recommended by Frank Harrel, with a 4-knots fitted at the 5^th^-, 35^th^, 65^th^, and 95^th^-quantile of the data for the ED occupancy levels and a 3-knots fitted at the 10^th^-, 50^th^- and 90^th^-quantile of the data for the hour of arrival to the ED and the month of arrival to the ED. The blue-shaded regions represent the 95% predicted intervals and the p-value for the association tested with a likelihood ratio test. The hour and month of arrival to the ED was also modelled but not presented as there were no visible effects on the outcome.
